# Dsg2 ectodomain organization increases throughout desmosome assembly

**DOI:** 10.1080/19336918.2024.2333366

**Published:** 2024-04-02

**Authors:** William F. Dean, Rose M. Albert, Tomasz J. Nawara, Melanie Ubil, Reena R. Beggs, Alexa L. Mattheyses

**Affiliations:** Department of Cell, Developmental, and Integrative Biology, University of Alabama at Birmingham, Birmingham, AL, USA

**Keywords:** Assembly, cadherin, cell-cell adhesion, desmosome, fluorescence polarization

## Abstract

Desmosomes are intercellular junctions that regulate mechanical integrity in epithelia and cardiac muscle. Dynamic desmosome remodeling is essential for wound healing and development, yet the mechanisms governing junction assembly remain elusive. While we and others have shown that cadherin ectodomains are highly organized, how this ordered architecture emerges during assembly is unknown. Using fluorescence polarization microscopy, we show that desmoglein 2 (Dsg2) ectodomain order gradually increases during 8 h of assembly, coinciding with increasing adhesive strength. In a scratch wound assay, we observed a similar increase in order in desmosomes assembling at the leading edge of migratory cells. Together, our findings indicate that cadherin organization is a hallmark of desmosome maturity and may play a role in conferring adhesive strength.

## Introduction

Desmosomes are macromolecular cell–cell junctions found predominantly in epithelia and cardiac muscle [1,2]. Desmosomes act primarily to maintain tissue integrity by forming strong intercellular adhesions, but they also play key regulatory roles during wound healing, tissue differentiation, and development [3–7]. Each desmosome is composed of hundreds of proteins from three distinct families. The desmosomal cadherins, desmogleins (Dsg) and desmocollins (Dsc), are calcium-dependent transmembrane proteins that comprise the adhesive core of the junction. Cadherin ectodomains from opposing cells form strong, hydrophobic *trans* interactions at the adhesive interface via the exchange of N-terminal β-strands. The cadherin cytoplasmic tails are binding scaffolds for the armadillo proteins, plakoglobin (PG) and plakophilin (Pkp), which, in turn, anchor the desmosome to intermediate filaments (IF) via the cytoskeletal adaptor, desmoplakin (DP) [2,6].

The assembly of constituent desmosomal proteins into mature junctions is incompletely understood, but the minimal set of components required is established and the dynamics of several assembly steps have been studied in detail [1,8–10]. Particularly well characterized are the events that occur on the scale of minutes immediately after the formation of adherens junctions, which include microtubule-dependent transport of Dsg2 and Dsc2 to sites of intercellular contact [11], recruitment of Pkp3 and Pkp2 to the plasma membrane [12], and translocation of DP into nascent junctions [8,12–14]. Less well understood are the subsequent, large-scale architectural changes that occur on the scale of hours as the desmosome matures, such as exclusion of E-cadherin [1], structural rearrangement of DP [15], narrowing of the distance between opposing plaques [2,15], and lengthening of the junction along the plasma membrane [15].

Through a variety of techniques, we and others have shown that cadherin ectodomains in mature desmosomes are organized into highly ordered assemblies [16–21], yet the relationship between cadherin architecture and desmosome function remains unclear. Given that the cadherins form the adhesive interface and provide a binding scaffold for plaque proteins, one possibility is that desmosomes rely on a precise arrangement of cadherins to supply adhesive strength and mechanical stability to the junction. In line with this idea, molecular dynamics simulations using atomic models derived from cryo-electron tomography (cryo-ET) experiments suggested that only certain arrangements of cadherin ectodomains could explain the unique biophysical features of desmosomal adhesion [17]. We recently demonstrated that cadherin ectodomain order is a phenomenon broadly conserved among multiple desmosomal cadherin isoforms – including Dsg3, Dsg2, and Dsc2 – further supporting the notion that cadherin architecture and desmosome function are intrinsically linked [16]. However, despite what is known about desmosome assembly, it remains unclear how and when cadherins attain this organization. Determining how cadherin architecture is acquired during assembly is crucial for understanding the relationship between cadherin organization and desmosome function, the mechanisms regulating adhesion during cell migration, and how adhesion is dysregulated in pathogenic states characterized by defective desmosome assembly.

Changes in desmosomal cadherin architecture are challenging to study due to the diffraction-limited size and compositional complexity of the desmosome. Desmosomal cadherins have a lateral spacing of roughly 7 nm [17], well below the Abbe diffraction limit [22], rendering traditional optical methods incapable of resolving subtle changes in cadherin organization. Conversely, while traditional structural methods like cryo-ET offer major improvements to spatial resolution, they provide limited information on protein specificity and dynamics [23], making them unsuitable to study architectural changes during assembly. Here, we overcome these technical challenges with excitation-resolved fluorescence polarization microscopy (FPM), which enables quantification of the ensemble order and orientation of fluorescently labeled molecules [24]. FPM is emerging as a useful tool to investigate the nanoscale architecture of the desmosome [16,25,26], as well as other biological assemblies that are difficult to study using traditional methods including the nuclear pore complex [27,28], yeast septins [29], integrins [30], and actin filaments [31].

**Figure 1.**
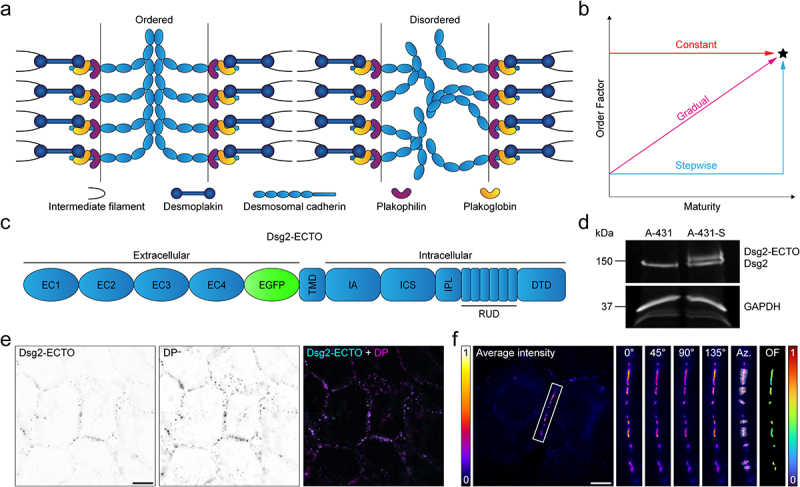
Measuring Dsg2 ectodomain order using excitation-resolved fluorescence polarization microscopy. (A) stylized schematic of a desmosome with either ordered (*left*) or disordered (*right*) cadherin ectodomains. (B) hypothesized pathways leading to the mature, ordered state of Dsg2 ectodomains (*black star*): constant (*red line*), gradual (*magenta line*), and stepwise (*cyan line*). (C) the construct used to measure Dsg2 ectodomain order, Dsg2-ECTO. (D) Western blot probing for Dsg2 in WT A-431 cells (*left*) and A-431 cells stably expressing Dsg2-ECTO (A-431-S, *right*) with a GAPDH loading control. (E) widefield fluorescence images of A-431 cells stably expressing Dsg2-ECTO (*left*) and immunolabeled for desmoplakin (DP, *middle*), with a merged image shown to the right. (F) excitation-resolved fluorescence polarization microscopy of Dsg2-ECTO in A-431-S cells. From left to right: the average intensity across all excitation polarizations with a representative cell border highlighted by a white, rectangular ROI, magnified ROIs showing the emission intensity at each excitation polarization, the azimuth (Az.) directions for each desmosome, and the order Factor (OF). All scale bars represent 10 μm.

In the present study, we investigate how Dsg2 ectodomain order (Figure 1A) is acquired during desmosome assembly initiated via two distinct triggers. First, we show that Dsg2 ectodomain order increases gradually over a period of 8 h during desmosome assembly initiated by a low-to-high calcium switch. The gradual increase in order was correlated with an increase in intercellular adhesive strength over the same time frame, suggesting cadherin organization might be related to adhesive function. Next, we initiated desmosome assembly using a scratch wound cell migration assay and observed a similar gradual increase in Dsg2 ectodomain order along individual cell borders leading away from the wound edge. Together, our findings reveal the manner in which cadherin order is acquired during desmosome assembly, paving the way for future studies of the relationship between desmosomal adhesion and the spatiotemporal dynamics of the desmosomal cadherins. Moreover, this study illustrates how FPM can be utilized to investigate the architectural dynamics of specific proteins within intricate complexes that are otherwise challenging to study using more traditional approaches.

## Materials and methods

### Dsg2 extracellular order probe and A-431-S cells

The Dsg2 extracellular order probe, Dsg2-ECTO, was made by inserting EGFP between V503 and L610 of human Dsg2 (UniProt: Q14126), as described previously [16]. Prior to generating the stable cell line, A-431 cells (ATCC, Manassas, VA) were cultured in Dulbecco’s Modified Eagle’s Medium (DMEM; Corning, Corning, NY) containing 10% fetal bovine serum (FBS) (Fisher Scientific, Waltham, MA). To generate an A-431 line stably expressing Dsg2-ECTO (A-431-S), cells were first transiently transfected with a plasmid encoding Dsg2-ECTO and the neomycin gene using Lipofectamine 2000 (Thermo Fisher Scientific, Waltham, MA) according to manufacturer’s instructions. After 6 h, the transfection medium was replaced with DMEM containing 10% FBS and 0.2 mg/mL geneticin (Fisher Scientific). Transfected cells were cultured for 3 weeks and then sorted using the BD FACSMelody to select for EGFP+ cells. After sorting, cells were cultured in DMEM containing 10% FBS and 0.2 mg/mL geneticin. All cells were maintained at 37°C and 5% CO_2_.

### Immunoblotting

A-431 and A-431-S cells were grown until confluent on a 6-well dish. To extract proteins, cells were first washed twice with 2 mL ice-cold PBS and then lysed in RIPA buffer supplemented with cOmplete (Roche 11697498001). The cells were then scraped and stored on ice for 30 min. The samples were forced through a syringe (BD Vacutainer 329622) three times and then centrifuged for 15 min at 12,000 RPM at 4°C. The supernatants were collected, and protein concentration was assessed using the Pierce BCA protein assay kit (Thermo Scientific 23227). Samples were loaded at 85 µg of total protein per well (Laemmli Sample Buffer) into an 8% SDS-polyacrylamide gel under denaturing conditions, and the size-fractionalized proteins were transferred to polyvinylidene difluoride membranes (Bio-Rad, Immun-Blot LF PVDF) overnight at 30 V and 4°C. Blots were blocked for 1 h at 4°C with Intercept Blocking Buffer (Li-Cor, 927-66003) and immunoblotted with primary antibodies overnight at 4°C, and secondary antibodies for 1 h at RT. Antibodies: rabbit anti-Dsg2 (Sigma, SAB2701981-100UL; 1:1000), mouse anti-GAPDH (Cell Signaling Technology 97166; 1:1000), goat anti-mouse IgG (Li-Cor, 925-68020, IRDye 680LT; 1:20,000) and goat anti-rabbit IgG (Li-Cor, 925–32211, IRDye 800CW; 1:15,000). All antibodies were diluted in Intercept Antibody Diluent (Li-Cor, 927-66003). Western blots were visualized using an Odyssey Image Station (Li-Cor) and the Odyssey Application Software (3.0, Li-Cor).

### Fixation

Prior to all imaging experiments, samples were fixed in a 1:1 solution of ice-cold acetone and methanol for 10 min at 4°C. Immediately after fixation, samples were washed with Phosphate Buffered Saline (PBS) three times, 5 min each, while gently shaking at RT. Samples were stored in PBS at 4°C. Immediately prior to imaging, coverslips were placed in AttoFluor chambers (Thermo Fisher Scientific) containing FluoroBrite DMEM (Thermo Fisher Scientific).

### Preparation of fibronectin-coated coverslips

Prior to each imaging experiment, 25 mm No. 1.5 glass coverslips (Thorlabs, Newton, NJ) were cleaned via sonication for 45 min in 200 proof ethanol. Coverslips were air-dried on parafilm and then coated for 45 min at RT with 250 µL of 25 μg/mL fibronectin in Hank’s Balanced Salt Solution (HBSS). After coating, coverslips were transferred into 6-well plates (Thermo Fisher Scientific) and rinsed three times with 1 mL HBSS.

### Calcium-free pulse assembly assay

Calcium-free FBS was prepared in 50 mL aliquots via chelation with 3.8 g of Chelex resin (Bio-Rad) for 1 h at 4°C while rotating. The FBS was then filtered through a Steriflip vacuum filter (Thermo Fisher Scientific). Calcium-free media were prepared using calcium-free DMEM (Corning), 10% calcium-free FBS, and 3 mM egtazic acid (EGTA). A-431-S cells were seeded onto fibronectin-coated coverslips and grown until confluent. For the calcium-free pulse, the coverslips were rinsed with 1 mL calcium-free media and then incubated in 2 mL calcium-free media for 45 min. The calcium-free media were then replaced with normal, calcium-containing DMEM with 10% FBS and 0.2 mg/mL geneticin. Cells were fixed at 2, 4, 6, and 8 h after restoring calcium. To act as controls, a ‘no-pulse’ sample was maintained in calcium-containing medium for the entire experiment and fixed along with the 8 h sample, while a ‘time 0’ sample was fixed immediately after the 45 min calcium-free pulse without restoring calcium.

### Immunofluorescence

A-431-S cells were grown until confluent on fibronectin-coated coverslips in DMEM containing 10% FBS and 0.2 mg/mL geneticin. Cells were fixed and washed 3 times, 5 min each, with PBS containing 0.5% Normal Horse Serum (NHS), 0.5% Normal Goat Serum (NGS), and 0.5% Triton X-100. After washing, samples were blocked for 30 min in PBS containing 5% NHS, 5% NGS, 0.5% Triton X-100, and 10 mg/mL bovine serum albumin (BSA). After blocking, cells were incubated with the primary antibody – rabbit anti-desmoplakin (Bethyl Labs, Montgomery, TX), diluted 1:150 in blocking buffer – for 1 h at 37°C in a humidity chamber shaking at 35 rpm. After primary incubation, samples were washed 3 times, 5 min each, and then incubated with the secondary antibody – Alexa647-conjugated goat anti-rabbit (Invitrogen), diluted 1:1000 in blocking buffer – for 30 min at 37°C in a humidity chamber shaking at 35 rpm. Immunolabeled samples were washed with PBS 3 times, 5 min each, and then imaged by standard widefield epifluorescence.

### Excitation-resolved fluorescence polarization microscopy

Excitation-resolved fluorescence polarization microscopy was performed using a Nikon-Ti2 microscope with a motorized stage and a 60✕, 1.49 NA oil-immersion objective. A continuous, 488 nm laser (Coherent, Santa Clara, CA) operating at 15 mW was passed through a linear polarizer (Thorlabs) and a digitally controlled, achromatic half-wave plate (AHWP10M-600; Thorlabs). The excitation beam was focused on the objective back focal plane with a focusing lens (Thorlabs). The emission was passed through an emission filter (ET525/50 m, Chroma) and captured on an ORCA-Flash 4.0 v3 complementary metal-oxide-semiconductor camera (Hamamatsu). The emission was collected in stacks of four images, 50 ms exposure each, using excitation polarizations of 0°, 45°, 90°, and 135° relative to the positive x-axis in the microscope coordinate system. To enable flat-field correction, three emission stacks were acquired at different locations on a flat, autofluorescent plastic slide (92001; Chroma), averaged along each excitation polarization, and then normalized to the maximum intensity in the stack.

### Image analysis

All image processing and analysis was conducted in MATLAB (The MathWorks, Natick, MA) using a custom software package (Object-Oriented Polarization Software), as described previously [16]. Briefly, the raw data were first flat-field corrected by dividing each element in the raw emission stack by the corresponding element in the flat-field stack. The images were then normalized pixel-by-pixel by dividing each set of four pixels by the maximum value across all excitation polarizations. The Order Factor (OF) and azimuth (α) were then calculated for each pixel:

$$A=I_{0}-I_{90}$$

$$B=I_{45}-I_{135}$$

$$\mathrm{OF}=\sqrt{A^{2}+B^{2}}$$

$$\alpha =\arctan2\left(B,A\right)/2$$

where $I_{0}$, $I_{45}$, $I_{90}$, and $I_{135}$ are the normalized, flat-field corrected intensities for each set of four pixels at the corresponding excitation polarizations, and ‘arctan2’ is the four-quadrant inverse tangent function.

To build image masks, we first generated an average intensity image by averaging each flat-field corrected stack across all excitation polarizations. The average intensity image was corrected for nonuniform illumination by applying a top-hat transform using a disk-shaped structuring element with a radius of 3. An intensity threshold was then automatically determined using Otsu’s method [32] and used to binarize the image. The resulting mask was then used to segment the image into objects, which were defined as individual connected components with 4-pixel connectivity. The object OF was defined as the mean OF for all of the pixels within each object. To quantify azimuthal disorder, we calculated the circular standard deviation of the azimuths in each object. Because azimuth values have π phase ambiguity, they were first transformed into complex unit vectors in the two-dimensional plane and mapped to a scale with a period of 2π, as has been described previously [33]. The mean resultant vector has the form:

$$\overset{ˉ}{r}=\frac{1}{N}\overset{N}{\underset{n=1}{\sum }}\exp\left(2i\alpha_{n}\right)$$

where N is the number of pixels within each object. The resultant vector length was then calculated by taking the norm of the mean resultant vector.

$$R=||\overset{ˉ}{r}||$$

Finally, the resultant vector length was used to calculate the circular standard deviation of the azimuths, and the data were unwrapped and mapped back to their original scale.

$$s_{0}=\frac{\sqrt{-2\ln R}}{2}$$

The local signal to background ratio (S/B) was determined by first creating a 3-pixel wide buffer zone that was excluded from the background. The local background area was defined as a two-pixel wide zone around the buffer, while the signal was defined as the area covered by each object mask. The local S/B was calculated by taking the ratio of the mean signal intensity in each region, and objects with an S/B < 3 were excluded from further analysis, as described previously [16].

### Dispase fragmentation assay

A-431-S cells were seeded into a 24-well plate and grown to confluence. Each monolayer was subjected to a 45 min calcium-free pulse and calcium was restored for 2, 4, 6, or 8 h, as described above. The no-pulse control was maintained in normal calcium throughout the experiment, while calcium was not restored for the time 0 control. After the calcium-free pulse assay, 1 U/mL Dispase II (Sigma-Aldrich) was added to each well for approximately 30 min – until the monolayer was entirely lifted from the plate. A P1000 tip was clipped and lubricated with FBS before subjecting each cell sheet to mechanical stress using a uniform number of pipette aspirations. The number of aspirations for each independent experiment was determined by the number required to produce countable fragments in the no-pulse control. After fragmentation, the samples were fixed with 1% paraformaldehyde (Electron Microscopy Sciences, Hatfield, PA) and fragments were manually counted under a dissection microscope. The assay was performed independently three times, with each experiment comprising three technical replicates per time point.

### Scratch wound cell migration assay

A-431-S cells were grown until confluent on fibronectin-coated coverslips in DMEM containing 10% FBS and 0.2 mg/mL geneticin. A P1000 pipette tip was used to produce a scratch across the center of the coverslip, and the medium was immediately replaced. After 2 h, samples were fixed and imaged with FPM. A brightfield image was captured of the same ROI to visualize the leading edge. To perform 2-dimensional OF line scans, a series of 18 parallel lines with a lateral spacing of 1 pixel were drawn along the cell border, for a final width of approximately 2 μm. Along each line, the MATLAB function ‘improfile’ was used to compute a line scan, and the scans were subsequently averaged along their normal direction to obtain the final line scan. Only pixels within the binary mask were included in the calculation. To compare the OFs between desmosomes flanking each cell border between leading edge cells, the desmosomes closest to the leading edge (group A) were compared to those farthest from the leading edge (group B). For confluent regions of the coverslip – because no leading edge is present – desmosomes flanking each cell border were randomly sorted into groups (group Aʹ or group Bʹ) prior to being compared.

### Statistical analysis

All statistical analysis was completed using Prism version 9 (GraphPad Software, San Diego, CA). Object OFs and azimuth circular standard deviations at each time point were compared using a Kruskal–Wallis test, followed by Dunn’s multiple comparisons test. The numbers of fragments counted at different time points in the dispase fragmentation assay were compared using an ordinary one-way ANOVA followed by Sidak’s multiple comparisons test. Technical replicates were pooled across all three dispase experiments for each timepoint prior to performing statistical comparisons. Object OFs for desmosomes flanking cell borders in the scratch wound assay were compared using a paired t-test. In all statistical analyses that involved multiple comparisons, data for each time point were only compared to neighboring time points and to the no-pulse control. With the exception of the paired t-test, all resulting *p* values were adjusted for multiple comparisons, with *p* < 0.05 considered statistically significant.

## Results

### Measuring Dsg2 ectodomain order using excitation-resolved FPM

We propose three distinct pathways that could lead to the mature, ordered state of cadherin ectodomains: constant, in which ectodomains are ordered early and remain ordered throughout junction maturation; gradual, where ectodomain order is steadily acquired during maturation; and stepwise, where cadherins are disordered until a conformational switch or signaling event triggers rapid ordering (Figure 1A,B). To study cadherin architecture changes during desmosome assembly, we chose Dsg2 as a representative protein because it is the only desmoglein expressed in all tissues that form desmosomes [1,5,6,34]. We were specifically interested in the Dsg2 ectodomain due to its role in forming the adhesive interface. An extracellular order probe for Dsg2, Dsg2-ECTO, was generated by replacing the extracellular anchor domain of Dsg2 with EGFP such that the EGFP transition dipole moment would reflect the orientation of the Dsg2 ectodomain (Figure 1C), as previously described [16].

To monitor cadherin organization in assembling desmosomes, we first generated A-431 cells stably expressing Dsg2-ECTO, which we denote A-431-S. Expression of endogenous Dsg2 and Dsg2-ECTO was confirmed by western blot (Figure 1D). Dsg2-ECTO localization to desmosomes was confirmed by immunolabeling for DP and imaging by widefield fluorescence. As expected, Dsg2-ECTO colocalized with endogenous DP in puncta along the cell borders (Figure 1E). Finally, we imaged A-431-S cells with excitation-resolved FPM (Figure 1F). FPM enables the retrieval of two key parameters: the Order Factor (OF), which represents the in-plane orientational order of all the EGFP dipoles within a single pixel; and the azimuth, which represents the average orientation of the projection of those dipoles onto the imaging plane. Because EGFP is constricted via its N- and C-terminal associations with Dsg2, OF and azimuth measurements of Dsg2-ECTO reflect the ensemble order and orientation of the Dsg2 ectodomain, respectively. As expected, both the OF and azimuth of Dsg2-ECTO in A-431-S cells were comparable to that seen in a prior study [16]. Together, these experiments demonstrate that Dsg2-ECTO was expressed and properly incorporated into desmosomes in A-431-S cells.

### Calcium-free pulse desmosome assembly assay

**Figure 2.**
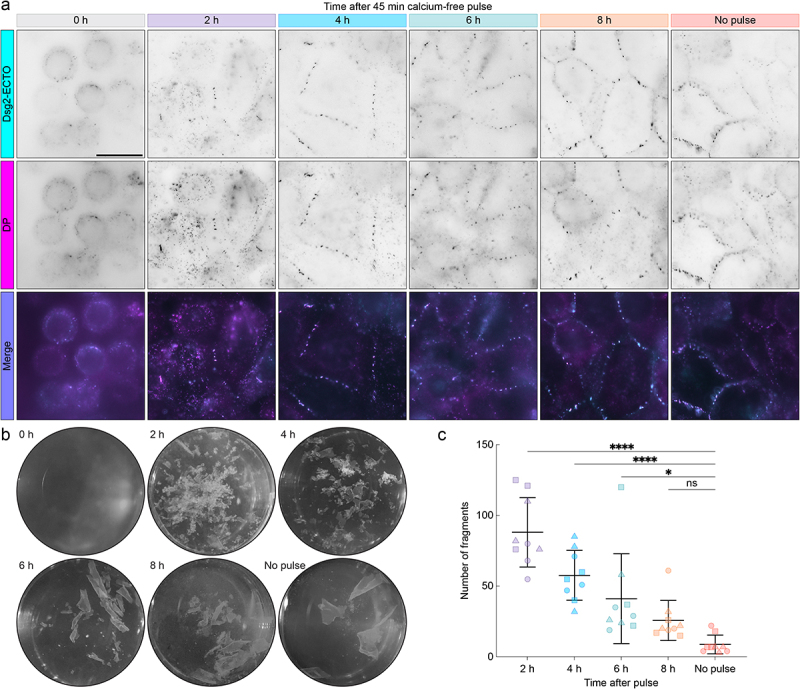
Dsg2-ECTO localizes to desmosomes without impacting the gain of adhesive strength. (A) Representative widefield fluorescence images of A-431-S cells stably expressing Dsg2-ECTO (*top row*) and immunolabeled for desmoplakin (DP, *middle row*) during the calcium-free pulse assembly assay. Merged images are shown in the bottom row. Top labels indicate the corresponding assembly time points. Scale bar represents 20 μm. (B) Representative images of A-431-S cells during a dispase fragmentation assay performed after the calcium-free pulse assembly assay. Image labels indicate the corresponding assembly time points. (C) quantification and statistical comparison of the number of fragments at each time point. Error bars indicate mean ± SD for data pooled across three independent experiments, each comprising three technical replicates per time point (*n* = 9; 2 h: 88.1 ± 24.5; 4 h: 57.7 ± 17.6; 6 h: 41.1 ± 31.8; 8 h: 25.8 ± 14.1; no-pulse: 8.8 ± 6.6). Plot markers corresponding to replicates from independent experiments are each shown as different shapes. Statistical significance was assessed with an ordinary one-way ANOVA followed by Sidak’s multiple comparisons test (****p < 0.0001; *p < 0.05; ns, *p* ≥ 0.05).

To study changes in Dsg2 ectodomain order during desmosome assembly, we synchronized the assembly process by manipulating extracellular calcium, similar to previous studies [9,10,35,36]. First, A-431-S cells were grown to confluence in normal, calcium-containing media. The cells were then switched into calcium-free media for 45 min, which led to rapid disruption of intercellular contacts (Figure 2A). After the 45 min calcium-free pulse, cells were switched into normal, calcium-containing media, thereby initiating desmosome assembly. Samples were fixed at 2, 4, 6, and 8 h following the restoration of calcium to establish an assembly timeline. A no-pulse control sample was maintained at physiologic calcium and a time 0 control sample was fixed immediately after the calcium-free pulse without restoring calcium. Samples were immunolabeled for DP and imaged by widefield to visualize the localization of Dsg2-ECTO to desmosomes during the assembly assay (Figure 2A). Importantly, in the time 0 control, cells displayed a rounded morphology with very few puncta localized to cell borders, indicating that the 45 min calcium-free pulse led to desmosome disassembly. Dsg2-ECTO colocalized with DP in puncta at cell borders beginning at 2 h, suggesting it was incorporated into desmosomes throughout the entire time course. Once we verified that Dsg2-ECTO localized properly throughout the assembly assay, we measured changes in intercellular adhesive strength over the time course. As anticipated, fragmentation following mechanical stress decreased with time, indicating an increase in adhesive strength (Figure 2B). By 8 h, fragment counts were not significantly different from those in control cells maintained in normal calcium media, suggesting adhesive strength had fully recovered (Figure 2C).

### Dsg2 order increases gradually during desmosome assembly

**Figure 3.**
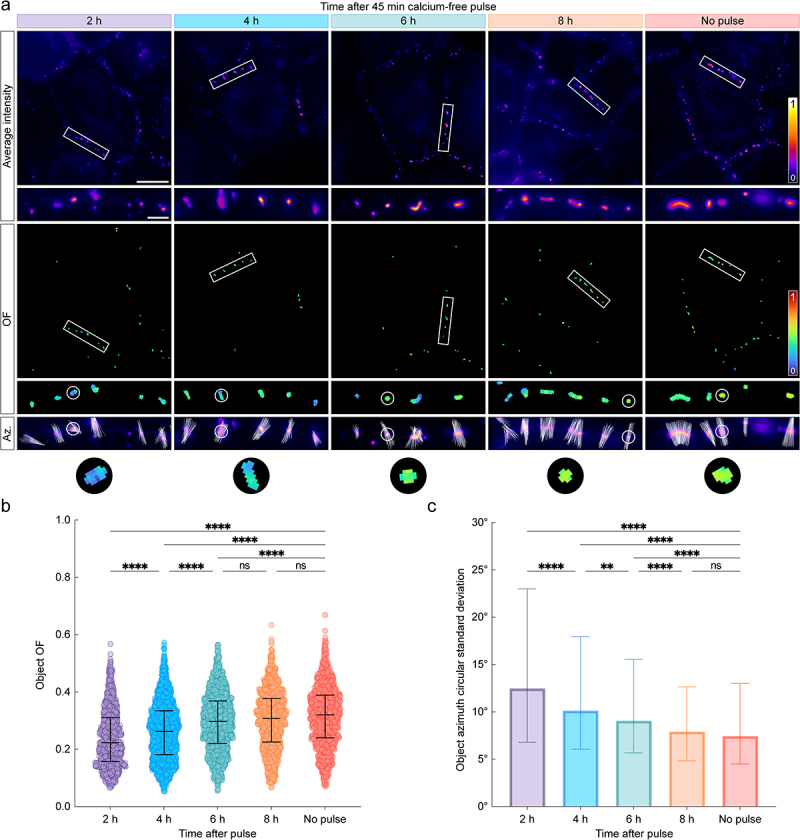
Dsg2 ectodomain order increases gradually during desmosome assembly. (A) normalized average intensity (*top*) and order factor (OF, *middle*) images of A-431-S cells acquired 2, 4, 6, or 8 h after a 45 min calcium-free pulse along with the no-pulse control. White rectangles indicate ROIs, which are shown magnified below their corresponding images. An additional set of ROIs is shown to illustrate the pixel azimuth directions (*white lines*) for each desmosome, where the length of each azimuth line is proportional to the of in its pixel. Scale bars represent 20 μm and 2 μm for full images and ROIs, respectively. (B) swarm plots showing the average of for each desmosome (object OF) across all experiments. Error bars represent median and interquartile range (IQR) (2 h: 0.22, IQR: 0.16–0.31, *n* = 1020; 4 h: 0.26, IQR: 0.18–0.33, *n* = 1813; 6 h: 0.30, IQR: 0.22–0.37, *n* = 1470; 8 h: 0.31, IQR: 0.23–0.38, *n* = 1258; no-pulse: 0.32, IQR: 0.24–0.39, *n* = 1108). (C) bar chart showing the circular standard deviation of the azimuth for each desmosome across all experiments. Error bars represent median and IQR (2 h: 12.49, IQR: 6.80–22.99, *n* = 1020; 4 h: 10.15, IQR: 6.07–17.96, *n* = 1813; 6 h: 9.08, IQR: 5.68–15.55, *n* = 1470; 8 h: 7.92, IQR: 4.83–12.65, *n* = 1258; no-pulse: 7.45, IQR: 4.50–13.02, *n* = 1108). For both B and C, statistical significance was assessed with a Kruskal–Wallis test followed by Dunn’s multiple comparisons test (****p < 0.0001; **p < 0.01; ns: not significant, *p* ≥ 0.05).

Next, we used the calcium-free pulse assembly assay to monitor changes in Dsg2 ectodomain organization during desmosome assembly. A-431-S cells were plated on glass coverslips, fixed, and imaged with excitation-resolved FPM at the pre-established timepoints (Figure 3). We observed that Dsg2-ECTO appeared to be less ordered during early assembly when compared to control; representative cell borders at each stage of assembly demonstrate a clear trend of increasing order (Figure 3A). To quantify the apparent increase in order, we calculated the average OF for each desmosome across all assembly timepoints. Beginning at 2 h, we observed a significant increase in OF until 6 h (Figure 3B). By 8 h, the median OF was not significantly different than the control. Notably, the gradual increase in OF is correlated with the gain of adhesive strength (Figure 2C), suggesting that proper cadherin architecture may be related to adhesive function. Importantly, while the distributions are broad, the mean OF observed in the no-pulse control (0.32 ± 0.10) is nearly identical to that observed in a previous study of the Dsg2 ectodomain (0.32 ± 0.11) [16], highlighting the precision of FPM in the quantification of ensemble architecture.

Next, we performed azimuthal orientation mapping to study the average orientation of the dipoles within each pixel, as projected into the sample plane. Previously, we showed that Dsg2-ECTO azimuths were consistently oriented orthogonal to the plasma membrane in mature desmosomes, indicating that cadherin ectodomains were arranged with rotational symmetry about the membrane normal [16]. By contrast, at early assembly timepoints, we observed that azimuths were not well aligned within individual desmosomes and did not show a strong preferential orientation with respect to the membrane. By 8 h, azimuths were well aligned within individual junctions, predominantly orthogonal to the plasma membrane, and not distinguishable from control junctions. To quantify this phenomenon, we calculated the azimuthal disorder for each desmosome, which we define here as the circular standard deviation of the azimuths within each junction ($s_{0}$) (Figure 3C). Unlike the OF, which reflects the relative alignment of the dipoles in a single pixel, the azimuthal disorder is inversely related to the alignment of pixel azimuths within each desmosome. Consistent with the increase in OF, we observed a significant, gradual decrease in azimuthal disorder that returned to control levels by 8 h. Together, our observations indicate that Dsg2 ectodomain architecture is acquired gradually over an 8 h period during calcium-induced desmosome assembly.

### Dsg2 order increases gradually along cell borders between migrating cells

While the calcium-free pulse assembly assay revealed a gradual increase in Dsg2 ectodomain order, we wondered whether assembly during a physiologically relevant process would yield similar results. It has been shown that a desmosome assembly gradient can be established using a scratch wound assay [37]. As cells migrate to heal the wound, desmosomes initially assemble at the leading edge. Nascent desmosomes then flow away from the wound in a retrograde fashion while increasing in size and maturity, leading to the establishment of an assembly gradient 1.5–2 h after wounding. To investigate how Dsg2 ectodomain order is acquired in assembling desmosomes between migratory cells, A-431-S cells were subjected to a scratch wound and allowed to migrate for 2 h before fixation and imaging with FPM.

**Figure 4.**
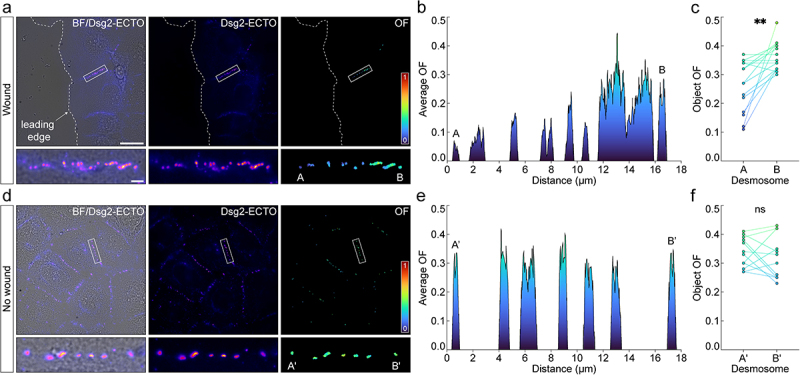
Dsg2 ectodomain order increases gradually along cell borders between migrating cells. (A) from left to right, brightfield (BF) with overlaid average intensity, average intensity, and order factor (OF) of A-431-S cells fixed 2 h after a scratch wound. The leading edge is labeled with a dashed white line. A representative cell border between leading edge cells is highlighted by a white rectangle and shown magnified beneath the corresponding images. Desmosomes closest to and farthest from the leading edge are labeled in the of ROI as ‘A’ and ‘B’, respectively. Scale bars represent 20 μm and 2 μm for full images and ROIs, respectively. (B) two-dimensional line scan between the desmosomes labeled in *A*, showing the change in average OF along the cell border. (C) paired dot plot showing the difference in OF between desmosomes closest to (‘A’) and farthest from (‘B’) the leading edge for multiple cell borders (means ± SD for *n* = 15 cell borders: A: 0.26 ± 0.09; B: 0.36 ± 0.05). Statistical significance was assessed with a paired t-test (**p < 0.01). *(D-F)* same as in *A-C* for cell borders on the same coverslip but located in fully confluent regions in the cell sheet, far from the leading edge. For each cell border analyzed, A′ and B′ represent desmosomes flanking the border, as depicted in the of ROI in *D* (means ± SD for *n* = 12 cell borders: Aʹ: 0.34 ± 0.05; Bʹ: 0.32 ± 0.07). Statistical significance was assessed with a paired t-test (ns: not significant, *p* ≥ 0.05).

We observed that order appeared to be lower in the immature desmosomes at the cell periphery and higher in the more mature desmosomes farther from the leading edge (Figure 4A). To visualize this phenomenon, we performed 2-dimensional OF line scans from the most nascent to the most mature desmosomes (Figure 4B). This revealed a clear, gradual increase in order along the assembly gradient. To quantify this observation, we measured the order of desmosomes closest to and farthest from the leading edge for multiple cell borders (Figure 4C). This revealed that immature desmosomes assembling at the leading edge were significantly less ordered than their more mature counterparts farther from the wound (Figure 4C). As a control, we examined cell borders on the same coverslip but located in fully confluent regions far from the wound (Figure 4D). Contrary to desmosomes assembling between migrating cells at the leading edge, desmosomes between non-migratory cells displayed consistent OFs along the entire border (Figure 4E). Likewise, the difference in OF between desmosomes flanking cell borders in confluent regions of the cell sheet was insignificant (Figure 4F). Taken together, our observations demonstrate that cadherin order is acquired gradually during desmosome assembly, whether initiated by changes in calcium or a scratch wound.

## Discussion

In this study, we investigated how cadherins become organized during desmosome assembly. First, we subjected cells to a calcium-free pulse followed by restoration of physiologic calcium to initiate synchronized desmosome assembly. We found that cadherin order in nascent junctions was low and steadily increased to control levels by 8 h, while azimuthal disorder steadily decreased to control levels over the same period, both indicating that cadherins become organized gradually as desmosomes mature. Next, we established an assembly gradient using a scratch wound migration assay. Consistent with our observations in the calcium-induced assembly assay, we observed a clear, significant increase in cadherin order along cell borders leading away from the wound edge, further supporting the notion that cadherin architecture is acquired gradually in assembling desmosomes.

Our study highlights the power of FPM in the study of ensemble architectural dynamics. Importantly, the approach taken here could be extended to a wide array of systems that are otherwise challenging to study using more traditional methods. Nevertheless, we faced several technical challenges worthy of further discussion. First, because the calcium-free media abolished all intercellular contacts, it is not possible to measure cadherin order at time 0. As a result, it remains unclear how our measurements at 2 h relate to cadherin architecture during the earliest stages of junction formation. Second, FPM measurements are highly sensitive to photobleaching. For that reason, while it would be ideal to track the order of individual assembling junctions in living cells, repeatedly imaging the same region for an extended period of time is not feasible. Third, our Dsg2 order probe was expressed in the presence of endogenously expressed cadherins. It remains unclear whether and to what extent these unlabeled cadherins may impact the observed architectural features. Finally, because there is not a technique capable of measuring the adhesive strength of individual junctions, it was not possible to directly link the observed architectural changes to desmosome function.

It remains unknown whether cadherins can become ordered on their own or rely on intracellular partners to facilitate the process. Notably, the discovery that Dsg2 ectodomain order is acquired gradually over hours contrasts with early steps in desmosome assembly, which occur on the scale of minutes upon initial cell contact formation [8,11,13]. While the exact mechanisms governing cadherin organization remain unclear, this behavior may reflect the impact of other large-scale architectural changes known to occur during desmosome maturation. For example, it has been shown that E-cadherin binds directly in *cis* to Dsg2 and is gradually excluded from assembling desmosomes [1]. It is possible that the presence of E-cadherin in immature junctions may prevent Dsg2 from properly organizing and that the increase in Dsg2 order reflects the concurrent exclusion of E-cadherin. Alternatively, it is possible that cadherin organization is governed by changes in the composition or spatial arrangement of plaque proteins over the course of maturation. Previously, we showed that ectodomain order was not significantly different between Dsg2 and Dsc2a, but that Dsc2b was significantly less ordered in mature desmosomes [16]. Dsc2a and Dsc2b share identical ectodomains, but Dsc2b lacks the full-length, PG-binding ICS domain [5], suggesting plaque proteins may play a role in organizing the cadherin ectodomains. Lastly, it is now well established that both intermediate filaments and cortical actin play key roles in facilitating early assembly [8,13,38]. Given the mechanical interplay between desmosomes and the cytoskeleton, it is also possible that cytoskeletal dynamics play a role in properly organizing the cadherins.

Importantly, the gradual increase in cadherin order observed here occurred alongside an increase in adhesive strength over the same time frame. Given that cadherins form the adhesive interface, it is tempting to conclude that proper cadherin architecture is required for desmosomal adhesive strength, as has been suggested previously [17,21]. However, because a direct link between cadherin organization and adhesive function has yet to be established, we consider several alternative explanations. First, it should be noted that the dispase fragmentation assay is not desmosome-specific; the degree of fragmentation is also affected by other intercellular junctions. Additionally, while we observed a consistent increase in ectodomain order, our FPM measurements are diffraction-limited and do not reveal how order is spatially distributed within individual junctions. It is possible that cadherin ectodomains in the core of the desmosome are in fact ordered early and remain highly ordered throughout assembly, while new cadherins at the periphery are less well ordered during their initial translocation into assembling junctions. In this scenario, as the total number of cadherins in the junction increases, each newly translocated molecule would make a smaller relative contribution to the observed OF.

Yet another possibility is that the increase in adhesive strength could be a function of the number of cadherins in each junction, rather than their progressive ordering. It could be reasonably argued that two desmosomes with identical cadherin geometries, but differing in the total number of cadherins, would react differently to mechanical stress due to the number of *trans* interactions comprising the adhesive interface. On the other hand, one might also predict that two desmosomes having the same number of molecules, but differing in the order of their cadherins, might also have different adhesive strengths. Indeed, molecular dynamics simulations of different cadherin arrangements showed that their resistance to externally applied force is influenced by their overall organization when the number of molecules is held constant [17]. Currently, it is not possible to completely disentangle the roles played by protein number and protein organization in conferring adhesive strength. It is possible that the adhesive strength of each junction depends on the synergistic effects of both the number of molecules and their organization.

Still, with regard to the relationship between structure and function in desmosomes, our work raises new, intriguing questions. Given the emerging understanding that cadherins are assembled into desmosomes in an isoform-dependent manner [11,39], it would be interesting to test whether the assembly behavior observed here for Dsg2 extends to other cadherin isoforms. Similarly, it remains unclear whether the architectural dynamics seen in A-431 cells extend more broadly to cell types in which different cadherin isoforms are expressed. A-431 cells predominantly express Dsg2 and Dsc2, with much lower levels of Dsc3 and Dsg3 [39,40]. Interestingly, it has been shown that cadherins can influence assembly dynamics irrespective of their relative expression levels [39]. Thus, the acquisition of cadherin order may differ in cell types such as cardiomyocytes and enterocytes, which only express Dsg2 and Dsc2 [41]. Likewise, while Dsg2 is broadly expressed across all desmosome-forming tissues [40], it is mostly absent from the suprabasal layers of stratified epithelia, whereas other isoforms such as Dsg3 are abundant [41]. In that sense, our findings may not directly apply to cell types with more complex cadherin expression profiles, such as normal keratinocytes. Investigating other cadherins and cell types may provide valuable insights into the complex interplay between different cadherin isoforms during desmosome assembly.

To our knowledge, this is the first investigation of cadherin architectural dynamics in assembling desmosomes. Together, our findings revealed the manner in which cadherins become organized during desmosome assembly and maturation, while providing novel insights into the relationship between cadherin ectodomain order and desmosome function. Furthermore, our work provides a framework by which future studies can examine the architectural dynamics of specific protein domains within larger complexes in a cellular context.

## Data Availability

The data that support the findings in this manuscript are available from the corresponding author, ALM, upon reasonable request.
